# Understanding the Drivers of Hypothyroidism in Patients Undergoing Chronic Hemodialysis

**DOI:** 10.3390/diagnostics16020177

**Published:** 2026-01-06

**Authors:** Ioana Adela Ratiu, Elena Emilia Babeș, Laura Monica Georgescu, Ozana Hocopan, Danut Dejeu, Corina Moisa, Daria Nicoleta Gavra, Cristian Adrian Ratiu

**Affiliations:** 1 Faculty of Medicine and Pharmacy, University of Oradea, 1st December Square 10, 410073 Oradea, Romania; ioana.ratiu@didactic.uoradea.ro (I.A.R.); eebabes@uoradea.ro (E.E.B.); lgeorgescu@uoradea.ro (L.M.G.); hocopan.ozana@rezident.uoradea.ro (O.H.); cristian.ratiu@didactic.uoradea.ro (C.A.R.); 2 Nephrology Department, Emergency Clinical Hospital Bihor County, 12 Corneliu Coposu Street, 410469 Oradea, Romania; 3 Cardiology Department, Emergency Clinical Hospital Bihor County, 65 Gheorghe Doja Street, 410169 Oradea, Romania; 4 Department of Medical Sciences, Endocrinology, Faculty of Medicine and Pharmacy, University of Oradea, 1st December Square 10, 410073 Oradea, Romania; 5 Surgery Department, Emergency Clinical Hospital Bihor County, 12 Corneliu Coposu Street, 410469 Oradea, Romania; 6 Department of Pharmacy, Faculty of Medicine and Pharmacy, University of Oradea, 1st December Square 10, 410073 Oradea, Romania; 7 Faculty of Medicine, “Iuliu Hațieganu” University of Medicine and Pharmacy, Victor Babeş Street, No. 8, 400012 Cluj-Napoca, Romania; gavra.daria.nicoleta@elearn.umfcluj.ro; 8 Dentistry Department, Discipline of Oral Implantology, Faculty of Medicine and Pharmacy, University of Oradea, 1st December Square 10, 410073 Oradea, Romania

**Keywords:** hypothyroidism, hemodialysis, subclinical hypothyroidism, amiodarone, cardiovascular mortality

## Abstract

**Background/Objectives**: Hypothyroidism is highly prevalent among HD patients, due to cumulative disturbances in thyroid hormone synthesis, metabolism, and clearance. Subclinical hypothyroidism—defined by elevated TSH with normal fT_4_—is common in HD, along with a distinct entity, the low-T_3_ syndrome. This study aims to examine the predictors of hypothyroidism in HD and its impact on cardiovascular morbidity and mortality. **Methods**: We conducted a retrospective cohort study including 282 hemodialysis (HD) patients, with evaluated thyroid function and monitored from January 2022 to June 2025. A total of 66 (23.4%) patients with hypothyroidism were identified, 15 (5.31%) of whom had autoimmune thyroiditis. Subclinical hypothyroidism was documented in 31.81% of the hypothyroid patients. **Results:** Hypothyroidism occurred predominantly in females (63.63% vs. 41.2%, *p* ≤ 0.001) and was associated with higher BMI (27.856 ± 6.216 vs. 25.759 ± 6.080, *p* = 0.017), hypoalbuminemia (3.534 ± 0.547 vs. 3.725 ± 0.471, *p* = 0.006), elevated LDL-cholesterol and triglyceride levels, as well as with amiodarone use. Hypothyroidism was further associated with atrial fibrillation (33.33 vs. 19.9%, *p* = 0.022), coronary artery revascularization procedures (18.18% vs. 9.72%, *p* = 0.047), neoplastic disease (25.75% vs. 12.03%, *p* = 0.008), and cancer-related mortality (10.6% vs. 1.85%, *p* = 0.001). Multivariable regression analysis revealed the following predictors of hypothyroidism: female sex (OR 3.848, 95%CI 1.704–8.693, *p* = 0.001), BMI (OR 1.072, 95%CI 1.007–1.146, *p* = 0.031), hypoalbuminemia (OR 0.412, 95%CI 0.177–0.962, *p* = 0.040), hypertriglyceridemia (OR 1.088, 95% CI 1.001–1.016, *p* = 0.022) and amiodarone use (OR 6.698, 95%CI 1.744–25.722, *p* = 0.006). Patients with autoimmune thyroiditis did not exhibit clinical or biochemical differences compared with other hypothyroid patients. Subclinical hypothyroidism was associated with longer HD duration (10.476 ± 7.910 vs. 6.567 ± 5.541, *p* = 0.003), dyslipidemia, hypertension, atrial fibrillation and amiodarone use. Cardiovascular conditions—particularly atrial fibrillation and ischemic coronary disease requiring revascularization—are more common in HD patients with clinical or subclinical hypothyroidism. However, in our cohort, the Kaplan–Meier survival curves at 12, 24, and 36 months for patients with both subclinical and clinical hypothyroidism do not show significant differences in cardiac or overall mortality. **Conclusions**: The increased incidence of hypothyroidism in HD patients, together with its impact on cardiovascular pathology, underscores the need for multidisciplinary management and supports annual routine assessment of thyroid hormones—particularly in overweight or dyslipidemic patients and in those receiving amiodarone.

## 1. Introduction

Hypothyroidism is a common condition among patients undergoing hemodialysis (HD), with a reported prevalence of 12.9% to 37.9% depending on geographic region [1,2,3,4,5,6,7,8]. It is associated with increased morbidity and mortality, primarily due to elevated cardiovascular risk resulting from systolic and diastolic dysfunction, endothelial dysfunction, and dyslipidemia that accelerates atherosclerosis [9]. In this regard, early diagnosis and timely correction of thyroid hormone imbalance are essential.

Hypothyroidism is classified as primary (resulting from thyroid hormone deficiency), secondary (due to inadequate thyroid-stimulating hormone production), tertiary (due to impaired thyrotropin-releasing hormone secretion), and peripheral (extra-thyroidal). In the general population, the most common form is primary hypothyroidism, which accounts for more than 99% of all cases [10].

In HD patients, as in the general population, hypothyroidism is mainly caused by autoimmune thyroiditis, nodular goiter, primary hypothyroidism, or secondary iatrogenic hypothyroidism, frequently linked to amiodarone use [11]. Hypothyroidism may be overt, characterized by elevated thyroid-stimulating hormone (TSH), abnormal free T_4_ (fT_4_), and characteristic clinical signs, or subclinical, defined by persistently elevated TSH with normal fT_4_ in the absence of apparent symptoms. Recognizing subclinical hypothyroidism as a distinct entity has emerged out of necessity due to its impact on morbidity and mortality [12,13]. The incidence of subclinical hypothyroidism is high in HD patients, ranging from 17.6% to 24.8%, but the impact of correcting hormonal imbalances through replacement therapy remains an area of ongoing research [14].

Hypothyroidism in HD patients arises from complex pathophysiological mechanisms, including altered thyroid hormone synthesis, metabolism, and impaired elimination due to reduced renal clearance and the use of dialysis membranes. Multiple pathological pathways contribute to this imbalance, including alterations in iodine metabolism via the Wolff–Chaikoff effect, protein–calorie malnutrition, chronic metabolic acidosis, uremic toxins, and selenium deficiency. Protein malnutrition markedly reduces T_4_ and T_3_ synthesis, as well as thyroid hormone-binding globulin and prealbumin levels. Reduced type 1(5′) deiodinase activity further impairs peripheral conversion of T_4_ to T_3_, resulting in lower circulating T_3_. Renal anemia reduces thyroid perfusion, and when combined with decreased dietary iodine intake due to sodium restriction, it impairs thyroid hormone synthesis. Uremia-associated electrolyte disturbances, including hypocalcemia and hyperphosphatemia, disrupt T_4_ secretion, whereas acidosis impairs ion channel function, further affecting thyroid hormone levels. Selenium deficiency, absence of renal clearance, and the pro-inflammatory state induced by dialysis also contribute to thyroid dysfunction.

The dialysis modality significantly impacts the risk of hypothyroidism: high-flux HD and hemodiafiltration are associated with a lower incidence by improving phosphate control, reducing inflammation, and enhancing T_4_-to-T_3_ conversion via superior clearance of uremic toxins [15]. Furthermore, heparin use and high-dose furosemide therapy inhibit thyroid hormone binding to protein substrates, promoting hypothyroidism.

In HD patients, hypothyroidism predominantly affects cardiovascular outcomes. It is associated with dyslipidemia and insulin resistance, promoting atherosclerosis, while bradycardia, arrhythmias, and structural cardiac changes—superimposed on pre-existing HD-related cardiac pathology—can trigger or exacerbate heart failure. In this context, hypothyroidism has been shown to increase both cardiovascular and all-cause mortality. Moreover, studies evaluating quality of life in HD patients with hypothyroidism have reported a comparative reduction across all domains [1,2,16].

Diagnosing hypothyroidism in a patient on HD is challenging, both from a clinical perspective and in terms of laboratory tests. Signs and symptoms of hypothyroidism are nonspecific in HD patients, in whom fatigue, pallor, tiredness, feeling cold, dry skin, edema, reduced body hair, muscle cramps, constipation, blood pressure changes, and rhythm disturbances are extremely common. From a paraclinical standpoint, measuring TSH, free T_3_ (fT_3_), and fT_4_ is not part of the routine investigations performed in HD patients; therefore, the diagnosis of thyroid dysfunction is most often incidental. Moreover, the cutoff values for TSH, fT_3_, and fT_4_ have been the subject of debate among patients undergoing HD. TSH is secreted by the pituitary gland and is regulated by circulating thyroid hormone levels (T_3_ and T_4_). Most of these hormones are protein-bound, primarily to thyroglobulin, transthyretin, and albumin, with less than 1% circulating in free form, which is biologically active at target tissues. In patients on HD, alterations in the synthesis, secretion, and clearance of these hormones make the interpretation of thyroid hormone levels—and consequently TSH—particularly challenging.

The latest guideline issued in 2014 by the American Thyroid Association (ATA) on hypothyroidism treatment addressed 24 key questions with evidence-based recommendations; however, these guidelines do not specifically address patients on HD. Similarly, the “Clinical Practice Guidelines for Hypothyroidism in Adults” by the American Association of Clinical Endocrinologists provides no specific recommendations for interpreting TSH, fT_3_, or fT_4_ in patients with HD. For a subclinical diagnosis, the elevation must be persistent over time, the hypothalamic-pituitary-thyroid axis must be intact, and severe consumptive illnesses must be absent. Evaluation of large, multiethnic, and demographically diverse patient cohorts has established a TSH cut-off value of 4.12 mIU/L [17]. However, given the distinctive clinical profile of patients undergoing HD and the continuously fluctuating blood composition, we emphasize the need to establish a dedicated set of recommendations, supported by relevant laboratory and clinical data, specifically tailored to this population.

A distinctive thyroid-related entity in patients undergoing HD is low T_3_ syndrome, characterized by reduced circulating triiodothyronine (T_3_) despite normal thyroxine (T_4_) and thyroid-stimulating hormone (TSH) concentrations [18,19]. TSH stimulates the release of fT_4_ and, to a lesser extent, fT_3_ from the thyroid gland. The circulating levels of fT_3_ primarily result from the peripheral conversion of fT_4_ to fT_3_ via 5′-deiodinase. In HD patients, reduced activity of this enzyme, coupled with increased clearance during dialysis, results in significantly lower fT_3_ levels. This phenomenon is driven by a complex interplay of factors associated with uremia, including malnutrition, metabolic acidosis, chronic inflammation, and the duration of HD. Consequently, the low T_3_ syndrome—also referred to as euthyroid sick syndrome or non-thyroidal illness syndrome—is extremely common in HD patients and typically occurs without an accompanying rise in TSH. It is widely interpreted as an adaptive response to the hypercatabolic state associated with uremia [18,19,20].

Management of hypothyroidism in HD patients is complex, raising questions regarding treatment of subclinical hypothyroidism, low fT_3_ syndrome, optimal TSH targets, levothyroxine dosing, and potential interactions with diet, medications, and dialysis [21,22]. Hormone replacement therapy aims to relieve symptoms and achieve normal TSH levels, ideally within the lower half of the reference range (0.5–2 mIU/L). Dose adjustments should be limited to 12.5–25 µg increments. TSH should be re-evaluated 4–6 weeks after initiation, at 6 months once target levels are achieved, and annually thereafter [17]. Potential drug interactions affecting thyroid hormone replacement therapy in HD patients include antacids such as aluminum hydroxide used for phosphate binding, proton pump inhibitors, sevelamer, certain beta-blockers such as propranolol, calcium-based supplements, and amiodarone, which exerts direct cytotoxic effects on thyroid follicles [21,22,23]. Amiodarone, due to its structural similarity to thyroid hormones, inhibits T4 deiodination, reducing T_3_ production and causing accumulation of T_4_ in the absence of its conversion to T_3_. Its administration results in more than a 40-fold increase in urinary and plasma iodine levels in individuals with normal renal function. Frequently used for the prevention and treatment of cardiac arrhythmias in HD patients, amiodarone also blocks T_3_ receptors and downregulates the expression of T_3_-dependent genes [23]. Although selenium has demonstrated antioxidant properties and some studies have reported reductions in anti-TPO antibody levels in autoimmune thyroiditis, there is currently insufficient evidence to support a beneficial role of selenium supplementation in the prevention or treatment of hypothyroidism [17].

Given the large number of monitored HD patients in our clinic, we aimed to assess the prevalence of all-forms of hypothyroidism, to identify predictors for its onset in HD patients and to evaluate the associated mortality.

## 2. Materials and Methods

We conducted a retrospective, observational cohort study that included 282 patients undergoing chronic HD, hospitalized between January 2022 and June 2025 at Bihor County Emergency Clinical Hospital. All patients provided a written informed consent at the time of hospitalization for the use of their personal data in clinical research. The study was conducted in accordance with the Declaration of Helsinki and was approved by the Bihor County Emergency Clinical Hospital Ethics Committee (Approval No. 24676/20 August 2025). Although the period covered in our retrospective study predates the ethics committee’s approval, the data were collected and processed after this approval.

The objectives of the study were

(a) To evaluate the prevalence of hypothyroidism in patients undergoing HD in a geographic area with a high incidence of thyroid disorders due to autoimmune thyroiditis and endemic goiter;
(b) To identify predictors of hypothyroidism in this population;
(c) To assess the impact of hypothyroidism on morbidity and mortality.

### 2.1. Study Design

**Figure 1:**
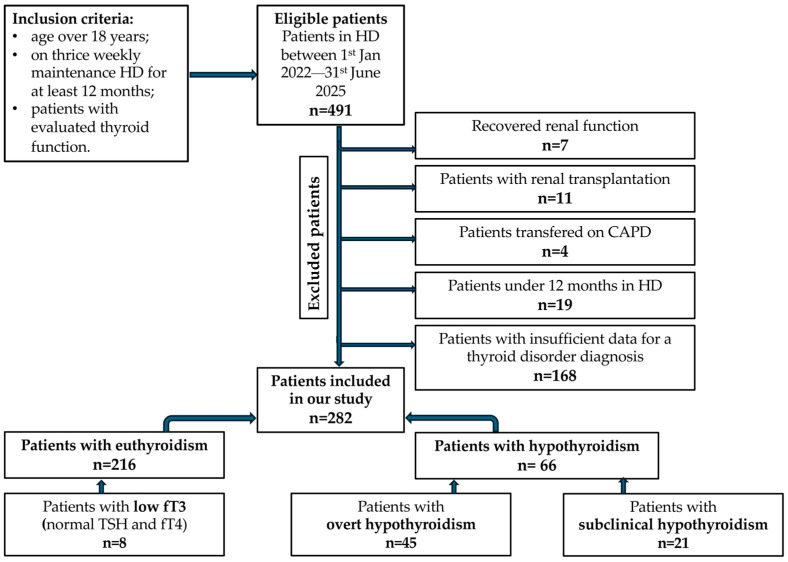
Flowchart outlining patient selection and inclusion into analysis groups.

We included 282 patients undergoing HD, followed between January 2022 and June 2025. Patients who initiated HD before the start of the study period and those with more recent HD initiation were included, provided they had at least 12 months of HD by the end of the observation period (Figure 1).

### 2.2. Inclusion Criteria

(a) Age > 18 years;
(b) On thrice-weekly maintenance HD for at least 1 year;
(c) data supporting the diagnosis of hypothyroidism.

### 2.3. Exclusion Criteria

(a) Missing key identifiers;
(b) Follow-up under 3 months due to kidney transplant or transfer to peritoneal dialysis.

### 2.4. Definitions

Overt hypothyroidism was defined as TSH > 4 mIU/L concomitant with fT_4_ and fT_3_ below laboratory reference limits.

Subclinical hypothyroidism was defined as TSH > 4 mIU/L without reductions in fT_4_ or fT_3_.

Autoimmune thyroiditis was diagnosed based on anti-thyroid peroxidase (anti-TPO) antibody titers exceeding 10 IU/mL.

In our laboratory, the reference range for fT_3_ was 1.88–3.18 pg/mL. Patients presenting with reduced fT_3_ levels, despite having normal TSH and fT_4_ values, were classified as euthyroid according to AACE guidelines. These patients were, therefore, included in the euthyroid cohort for further analysis.

All patients previously diagnosed with hypothyroidism receiving hormone replacement therapy or under monitoring were included in the hypothyroidism group. The majority of them, 36 out of 66, have been diagnosed prior to the study window: 30 patients had overt hypothyroidism, and 6 had subclinical hypothyroidism. During the study window, 15 patients were diagnosed with overt hypothyroidism and 15 patients were diagnosed with subclinical hypothyroidism.

Data related to hormone replacement therapy were insufficient, available only for 37 patients. 29 out of 30 patients diagnosed with overt hypothyroidism before the study window, had a documented treatment with levothyroxine, with a mean dose of 67.096 ± 39.425 (ranging from 12.5 to 150 micrograms). In terms of the patients who entered the study after its initiation time, data regarding replacement therapy was available for 8 patients, with doses ranging from 25 to 75 micrograms and a mean of 34.375 ± 18.6.

### 2.5. Data Sources and Collection

The data was collected from hospital admission records and the hospital’s electronic databases, hospital discharge summaries, and specialized outpatient clinic documentation, and included the following:

(a) **Demographic and biometric data:** age, sex, body mass index (BMI).
(b) **Dialysis data:** dialysis vintage (years), dialysis adequacy (Kt/V), vascular access (arteriovenous fistula/central venous catheter). During the study period, HD was performed using Braun Dialog + (Melsungen, Germany) and Fresenius 4008S (Bad Homburg vor der Höhe, Germany) machines. Water for dialysis was supplied by purification systems ensuring a bacterial count below 0.1 CFU/mL. Polysulfone membrane dialyzers were used, including Elisio (Nipro, Osaka, Japan), FX (Fresenius, Bad Homburg, Germany), and Diacap Pro (Braun, Kronberg, Germany), with surface areas of 1.9–2.1 m^2^ and ultrafiltration coefficients (Kuf) of 75–82 mL/h/mmHg, sterilized by gamma irradiation, and Helix One dialyzers with surface areas of 1.8–2.2 m^2^, Kuf 53–68 mL/h/mmHg, sterilized by inline steam at 121 °C for 15 min. The recommended target Kt/V for patients on thrice-weekly HD was set between 1.2 and 1.4.
(c) **Underlying kidney disease:** chronic glomerulonephritis (CGN), autosomal dominant polycystic kidney diseases (ADPKD), vascular nephropathies (VN), diabetic nephritis (DN).
(d) **Laboratory parameters:** hemoglobin (Hb), albumin, total cholesterol, LDL-cholesterol, HDL-cholesterol, triglycerides, calcium, phosphate, magnesium, high specific C reactive protein (hs-CRP), intact parathyroid hormone (iPTH), NT-proBNP. Biochemical parameters were measured using the chemiluminescent microparticle immunoassay (CMIA) technique on the Abbott Alinity C (AC06028) analyzer, while hemoglobin levels were assessed using CMIA on the Abbott Alinity hq (HQ00687) analyzer; NT-proBNP (reference range: 10.5–125 pg/mL) was measured using a chemiluminescent microparticle immunoassay (CMIA) on the Abbott Alinity c analyzer (AC03837).
(e) **Thyroid function:** TSH, fT_3_, fT_4_, anti-thyroid peroxidase antibodies (anti-TPO) were assessed using the CMIA technique on Alinity Ac03837 analyser, Abbott Park, IL, USA.
(f) **Comorbidities:** arterial hypertension, diabetes mellitus, neoplastic diseases, stroke, coronary artery disease (CAD), myocardial infarction (MI), percutaneous coronary intervention (PCI), paroxysmal atrial fibrillation (AF), permanent AF, acute cardiac failure.
(g) **Medication use:** amiodarone.

All analyses were performed using a single-landmark design, with one landmark time per patient. Continuous variables are presented as mean ± standard deviation (SD), and categorical variables as counts and percentages. Comparisons between groups were conducted using the independent samples *t*-test for continuous variables and the Chi-square test for categorical variables. A *p*-value < 0.05 was considered statistically significant. Variables that demonstrated significant differences were further evaluated using multivariable binary logistic regression to assess their independent predictive value. We performed univariate survival analyses for overt and subclinical hypothyroidism at 12, 24, and 36 months of follow-up, and generated survival curves based on Kaplan–Meier estimates. Statistical analyses were conducted using Jamovi version 2.7.2 and verified in SPSS version 25 (IBM Corp., Armonk, NY, USA).

## 3. Results

### 3.1. Hypothyroidism

#### 3.1.1. Clinical and Laboratory Characteristics of Patients with Hypothyroidism

Patients in HD with hypothyroidism were comparable with euthyroid individuals in terms of age (63.712 ± 14.103 vs. 64.444 ± 14.13, *p* = 0.707) and dialysis vintage (6.985 ± 6.487 vs. 6.648 ± 5.717, *p* = 0.678). Arteriovenous fistula was the predominant vascular access modality in both groups (*p* = 0.237). Hypothyroidism was more prevalent in female patients (63.63% vs. 41.2%, *p* ≤ 0.001) and body mass index (BMI) was significantly higher in hypothyroid patients (27.856 ± 6.216 vs. 25.759 ± 6.080, *p* = 0.017). There were no differences between groups regarding the underlying renal disease.

Laboratory analyses demonstrated that hypothyroid patients had significantly lower serum albumin (3.534 ± 0.547 vs. 3.725 ± 0.471, *p* = 0.006) and a dyslipidemic profile, characterized by elevated levels of total cholesterol (167.5 ± 55.291 vs. 151.0 ± 43.848, *p* = 0.042), LDL-cholesterol (114.375 ± 42.096 vs. 95.834 ± 36.697, *p* = 0.002), and triglycerides (175.258 ± 53.984 vs. 133.061 ± 59.761, *p* ≤ 0.001), while HDL-cholesterol levels were similar (36.402 ± 10.074 vs. 39.593 ± 19.214, *p* = 0.231). No significant differences were observed in anemia severity (hemoglobin 10.439 ± 1.311 vs. 10.656 ± 1.490, *p* = 0.228), inflammatory status (hsCRP 30.111 ± 38.465 vs. 28.688 ± 44.657, *p* = 0.817), calcium-phosphate balance or iPTH levels (412.07 ± 436.054 vs. 405.425 ± 436.702, *p* = 0.921).

Hypothyroid patients exhibited lower dialysis adequacy (Kt/V 1.512 ± 0.289 vs. 1.596 ± 0.312, *p* = 0.053) and a significantly higher frequency of amiodarone use (19.69% vs. 3.24%, *p* < 0.001). Overall mortality did not differ between hypothyroid and euthyroid patients (57.57% vs. 56.48%, *p* = 0.875) (Table 1).

#### 3.1.2. Comorbidities and Mortality Causes in Hypothyroidism

The incidence of hypertension, diabetes mellitus, coronary artery disease (CAD), heart failure, and stroke did not differ significantly between hypothyroid and euthyroid patients. Statistically significant differences were observed for the prevalence of atrial fibrillation (33.33 vs. 19.9%, *p* = 0.022) and the need for interventional cardiac procedures (18.18% vs. 9.72%, *p* = 0.047). Additionally, hypothyroid patients demonstrated a higher prevalence of neoplastic disease (25.75% vs. 12.03%, *p* = 0.008) and cancer-related mortality (10.6% vs. 1.85%, *p* = 0.001) (Table 2).

**Table 2:** Comorbidities and mortality causes in hypothyroidism.

Parameter	Entire Cohort 282 M ± SD/%	Hypothyroidism 66 (23.4%) M ± SD/%	Euthyroidism 216 (76.55%) M ± SD/%	*p*
Hypertension	265 (93.97%)	60 (90.90%)	205 (94.9%)	0.083
Diabetes	89 (31.56%)	17 (25.75%)	72 (33.33%)	0.220
CAD	91 (32.26%)	21 (31.81%)	70 (32.4%)	0.874
AHF	131 (46.45%)	25 (37.87%)	106 (49.07%)	0.150
Stroke	33 (11.7%)	7 (10.6%)	26 (12.03%)	0.734
Malignances	43 (15.24%)	17 (25.75%)	26 (12.03)	0.008
ICADR	33 (11.7%)	12 (18.18%)	21 (9.72%)	0.047
Paroxysmal AF	97 (34.39%)	17 (25.75%)	80 (37.03%)	0.091
Permanent AF	65 (23.04%)	22 (33.33%)	43 (19.9%)	0.022
CV death	78 (27.65%)	13 (19.69%)	65 (30.09%)	0.098
Stroke death	11 (3.9%)	2 (3.03%)	9 (4.1%)	0.676
Neoplastic death	11 (3.9%)	7 (10.6%)	4 (1.85%)	0.001

Legend: CAD—coronary artery disease; AHF—acute heart failure; ICADR—interventional coronary artery disease revascularization; AF—atrial fibrillation; CV—cardiovascular.

**Figure 2:**
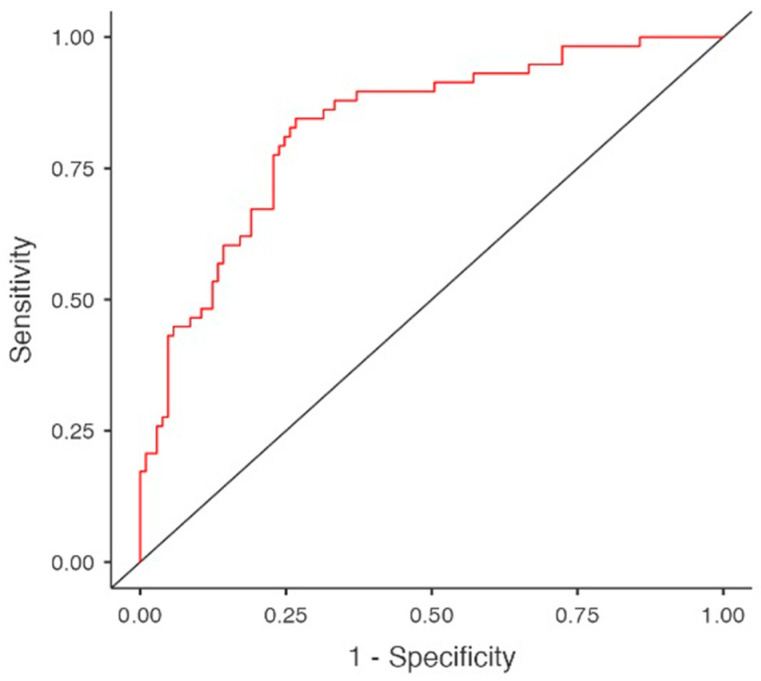
Predictors for hypothyroidism in multivariable regression model. Area under curve (AUC).

Multivariable logistic regression, yielding a model with a sensitivity of 0.569, specificity of 0.856 and an area under the curve (AUC) of 0.822, did not confirm a definitive association between neoplastic diseases and hypothyroidism. In this predictive model, hypothyroidism was significantly associated with female gender (OR 3.848, 95%CI 1.704–8.693, *p* = 0.001), BMI (OR 1.072, 95%CI 1.007–1.146, *p* = 0.031), hypoalbuminemia (OR 0.412, 95%CI 0.177–0.962, *p* = 0.040), hypertriglyceridemia (OR 1.088, 95% CI 1.001–1.016, *p* = 0.022) and amiodarone use (OR 6.698, 95%CI 1.744–25.722, *p* = 0.006), (Table 3 and Table 4, Figure 2).

The multivariable regression analysis was performed in an exploratory manner to identify potential associations and should be interpreted as hypothesis-generating rather than confirmatory.

**Figure 3:**
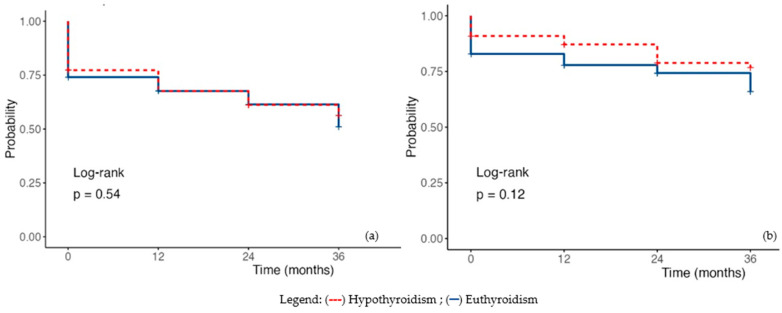
Kaplan–-Meier Survival Plot in hypothyroidism (**a**) Overall mortality; (**b**) Cardiac mortality. Legend: (---) subclinical hypothyroidism; (—) euthyroidism.

Using univariate regression to estimate survival at 12, 24, and 36 months, patients with hypothyroidism did not demonstrate statistically significant differences compared with euthyroid individuals (HR 0.88, 95%CI 0.58–1.33, *p* = 0.533). Analysis of cardiovascular-related mortality confirmed these findings (HR 0.62, 95%CI 0.32–1.13, *p* = 0.119) (Figure 3a,b).

#### 3.1.3. Autoimmune Thyroiditis in HD Patients

When isolating the subgroup of patients with hypothyroidism due to autoimmune thyroiditis and comparing them with the remaining hypothyroid patients, the only statistically significant difference observed was BMI (31.067 ± 5.951 vs. 27.856 ± 6.216, *p* = 0.022). Age and dialysis vintage did not differ between the groups, and females predominated in both. Laboratory parameters, including anemia, dyslipidemia, inflammatory status, and calcium-phosphate balance, were similar. No differences were observed regarding cardiovascular or neoplastic comorbidities, nor in mortality associated with these conditions. Therefore, the hypothyroid patient cohort appears homogeneous, regardless of the underlying etiology of hypothyroidism (Table 5).

### 3.2. Subclinical Hypothyroidism in HD

Subclinical hypothyroidism was associated with a longer duration on HD (10.476 ± 7.910 vs. 6.648 ± 5.717, *p* < 0.001), without differences in age (63.286 ± 14.364 vs. 64.444 ± 14.13, *p* = 0.360), sex (61.9% vs. 41.29%, *p* = 0.109), BMI (*p* = 0.466) or underlying renal disease compared with the euthyroid population. Significant differences were found in albumin level (3.51 ± 0.618 vs. 3.725 ± 0.471, *p* = 0.026) and TSH (4.647 ± 1.785 vs. 2.065 ± 1.209, *p* < 0.00001). Hemoglobin (10.519 ± 1.086 vs. 10.652 ± 1.461, *p* = 0.683), calcium, phosphate and iPTH levels were likewise comparable between the two groups.

Regarding the lipid profile, statistically significant differences were observed in triglyceride (178.048 ± 11.436 vs. 133.061 ± 59.761, *p* = 0.0005) and LDL-cholesterol levels (117.905 ± 51.103 vs. 95.834 ± 36.697, *p* = 0.0091). Patients with subclinical hypothyroidism also had lower serum magnesium levels compared with euthyroid patients (*p* > 0.05).

Among those with subclinical hypothyroidism, the incidence of chronic atrial fibrillation was higher (52.38% vs. 19.9%, *p* = 0.0018), as was the use of amiodarone (23.8% vs. 3.24%, *p* = 0.003). Cancer-related mortality was also more frequent in this group (14.28% vs. 1.85%, *p* = 0.011) (Table 6).

**Figure 4:**
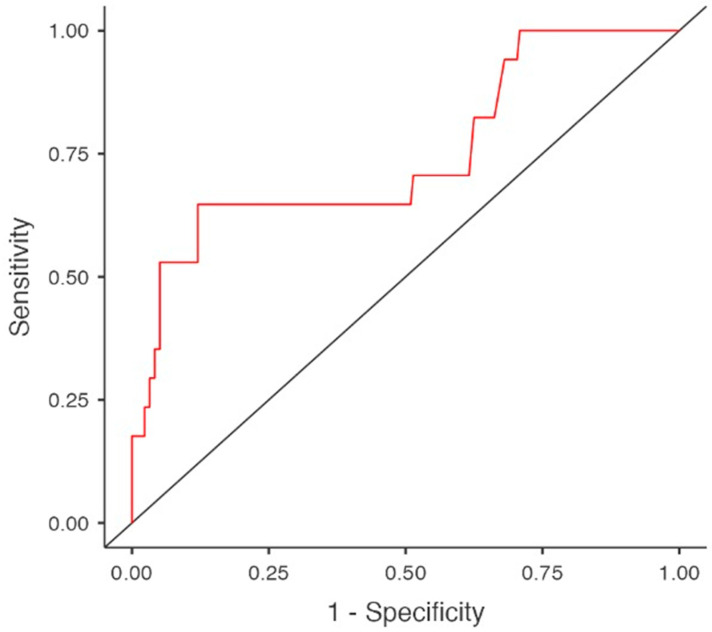
Predictors for subclinical hypothyroidism in multivariable regression model. Area under curve (AUC).

Given the limited number of hypothyroidism cases (*n* = 22), the multivariable logistic regression model was restricted to a maximum of three variables, selected based on clinical and biological relevance, in order to minimize the risk of model overfitting. The model incorporated HD vintage, serum albumin levels—considered to be less influenced by pharmacological interventions—and amiodarone use. The analysis demonstrated a substantial impact of both HD duration (OR 1.093, 95%CI 1.016–1.175, *p* = 0.017) and amiodarone exposure (OR 13.649, 95%CI 3.536–52.691, *p* < 0.001) on the development of subclinical hypothyroidism. Although serum albumin levels were significantly lower in the linear regression analysis, they did not emerge as an independent predictor of hypothyroidism in the selected multivariable regression model (OR 0.380, 95%CI 0.139–1.041, *p* = 0.06) (Table 7 and Table 8, Figure 4).

**Figure 5:**
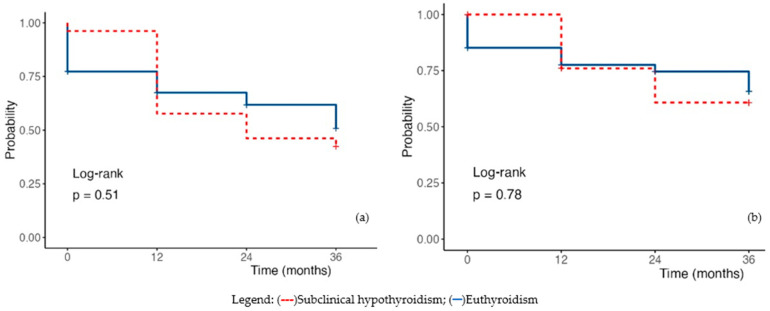
Kaplan-Meyer survival plot (**a**) Overall mortality; (**b**) Cardiac mortality. Legend: (---) subclinical hypothyroidism; (—) euthyroidism.

The Kaplan–Meier survival curve for patients with subclinical hypothyroidism does not show statistically significant differences in overall mortality (HR 1.18, 95%CI 0.69–2.03, *p* = 0.550). The analysis of cardiovascular-related mortality further confirms these findings, demonstrating a similar statistical association (HR 1.09, 95%CI 0.52–2.18, *p* = 0.818) (Figure 5).

### 3.3. Impact of Amiodarone on the Development of Hypothyroidism

A separate analysis comparing patients treated with amiodarone to those not receiving this medication demonstrates its impact on the incidence of hypothyroidism. This finding is notable given that no statistically significant differences were observed between the two groups in terms of age (68.200 ± 11.143 vs. 64.031 ± 13.875, *p* = 0.191), dialysis vintage (7.2 ± 5.899 vs. 6.599 ± 5.598, *p* = 0.645), type of vascular access, or underlying renal disease. However, BMI in patients receiving amiodarone was significantly higher (28.5 ± 5.066 vs. 26.056 ± 6.240; *p* = 0.025). Laboratory parameters—including hemoglobin (10.319 ± 1.031 vs. 10.642 ± 1.476, *p* = 0.337), albumin (3.526 ± 0.501 vs. 3.694 ± 0.498, *p* = 0.147), inflammatory markers (24.725 ± 37.855 vs. 29.097 ± 43.293, *p* = 0.662), and calcium–phosphate balance—were likewise similar in both groups, as was the lipid profile, with similar values for LDL- cholesterol (96.5 ± 41.458 vs. 102.6 ± 42.068, *p* = 0.608) and triglycerides (148.647 ± 87.491 vs. 149.265 ± 95.597, *p* = 0.980). Dialysis adequacy did not differ between amiodarone users and non-users (1.516 ± 0.289 vs. 1.580 ± 0.312, *p* = 0.398).

Used primarily for the management of recurrent paroxysmal atrial fibrillation, amiodarone was significantly associated with a higher incidence of hypothyroidism among patients undergoing HD (65% vs. 2.72%, *p* < 0.001) (Table 9).

## 4. Discussion

### 4.1. The Prevalence of Hypothyroidism in HD Patients

The prevalence of hypothyroidism in the general population is estimated at 5–7%, depending on geographic region; however, recent meta-analyses indicate that an additional 5% should be accounted for, representing previously undiagnosed hypothyroidism [24]. Hypothyroidism in HD patients has been investigated over time in various studies, across different population groups and from multiple perspectives. Several studies have reported the prevalence of hypothyroidism in HD, generally ranging between 18 and 28%, depending on geographic region: 12.8–18.6% in Iran [25,26], 20.3 in Italy [27], 20.6% in Pakistan [6], 21.8% in Brazil [28], 24.4% in Romania [11] and 28% in Somalia [5]. In Rhee’s study (2013), the incidence of hypothyroidism was 12.8% [1], whereas in the largest cohort reported by the same author, the prevalence was estimated at 22% [2]. Smaller, single-center studies conducted in Saudi Arabia report significantly lower incidences of hypothyroidism, around 5.1% [29]. Subclinical hypothyroidism accounts for less than 50% of the previously mentioned percentages. In our cohort, the incidence of hypothyroidism was 23.4%, consistent with data reported in the literature. It should be noted that the cohort was selected from an iodine-deficient region, endemic for nodular goiter, with a high prevalence of Hashimoto’s thyroiditis; the incidence of the latter in our cohort was 22.72%.

Although the NHANES III study demonstrated a twofold increased risk of hypothyroidism in patients with eGFR < 30 mL/min compared with those with normal eGFR, in HD patients, uremia masks the typical symptoms of hypothyroidism, making the diagnosis more difficult in the absence of a relevant clinical presentation [30]. Although TSH measurement remains the cornerstone for confirming hypothyroidism, its value may fluctuate substantially as a result of circadian and seasonal biological rhythms, advancing age, increased body weight, smoking, acute illness, environmental exposures, and the influence of various medications. Moreover, because TSH is released in pulses, even measurements taken minutes apart can differ. Therefore, interpreting TSH levels requires caution and clinical context [31]. In HD patients—who are predominantly elderly, overweight, burdened with multiple comorbidities and often subjected to complex therapeutic regimens—the interpretation of TSH values becomes challenging. Since our aim was not to assess the impact of correcting hypothyroidism, we included in the hypothyroid group those patients already receiving hormone replacement therapy. Even under these conditions, the mean TSH level recorded was significantly higher in the hypothyroid group compared with the euthyroid group, exceeding 10 mIU/L—a value that unequivocally confirms the diagnosis of hypothyroidism. Although reduced, fT_4_ levels did not differ significantly from those of euthyroid HD patients, whereas fT_3_ levels were markedly decreased in the hypothyroid group.

In HD patients, the low T_3_ syndrome is thought to represent a compensatory response of the body to the hypercatabolic state caused by uremia. However, the DREAM study demonstrated an association between this syndrome and both cardiovascular disease and all-cause mortality [19,32]. A meta-analysis of 12 studies indicated that low T3 is associated with a hazard ratio of 1.67 (95% CI, 1.23–2.27) for all-cause mortality and 1.84 (95% CI, 1.24–2.74) for cardiovascular mortality [33]. Other studies show that T3 predicts MACE only when considered alongside proBNP and existing cardiovascular disease [32]. fT_3_ has well-established cardioprotective effects, reducing oxidative stress, inflammation and apoptosis, stabilizing mitochondria, and protecting genetic material from pathogenic mutations. It regulates the chronotropic, dromotropic, and inotropic activity of the heart, both by controlling myocardial collagen content and by influencing transmembrane transport of ions, glucose, and amino acids in the myocardium. In patients with heart failure and low fT_3_, short-term fT_3_ supplementation has been shown to improve cardiac performance [34]. Under these circumstances, TSH is regarded as the most sensitive and specific single marker for hypothyroidism in the general population due to its inverse logarithmic relationship with circulating thyroid hormones (T_3_ and T_4_). Consequently, it is the preferred parameter for screening, diagnosis, and monitoring the treatment of primary hypothyroidism.

### 4.2. Characteristics of HD Patients with Hypothyroidism and Predictors of Hypothyroidism

In the general population, hypothyroidism primarily affects individuals over 65 years of age, more frequently females and is often associated with autoimmune conditions such as type 1 diabetes, celiac disease, or multiple endocrine autoimmunity [35,36,37,38]. The HD population is generally older, with an average age above 60 years globally, and this trend is increasing [39,40,41,42]. In our cohort, the mean age was 64.27 years, with hypothyroidism detected at slightly younger ages compared to euthyroid patients. Similarly, in a cohort from India, the mean age was 43 years, whereas in studies from Europe and the USA [1,11,14,43] it ranged from 54 to 64.7 years. In our patients, hypothyroidism occurred predominantly in females (63.63%), both in autoimmune cases (60%) and in non-autoimmune induced hypothyroidism (64.7%). These findings are consistent with data from the literature on hypothyroidism epidemiology in the general population, as well as with some studies conducted in HD patients [11]. However, in most large-scale studies, the proportion of male HD patients with hypothyroidism exceeds 50% [1,14,43].

In the general population, hypothyroidism is more common in overweight or obese individuals, with obesity acting both as a cause and a consequence of the condition [44]. Studies have shown that in morbid obesity, TSH levels are elevated relative to fT_4_ levels when compared with the non-obese population, potentially due to increased leptin synthesis [45]. Evidence from the literature indicates that the majority of HD patients with hypothyroidism are overweight, although instances of patients with normal body weight have also been documented [11,14,16]. The biometric profile of our patients was characterized by being overweight. Compared with the overall cohort mean of 26.292 ± 6.171, the BMI in patients with hypothyroidism was 27.856 ± 6.216 (*p* = 0.017).

The underlying renal disease was predominantly chronic glomerulonephritis, while vascular nephropathy was less frequent. No statistically significant differences were observed regarding the cause of CKD between hypothyroid and euthyroid patients. The duration of HD in our patients was comparatively longer than in other cohorts evaluating this pathology, nearly 7 years. HD vintage was positively correlated with the prevalence of hypothyroidism, but only in the case of subclinical hypothyroidism. However, multivariate regression did not identify this parameter as a significant predictor in the proposed model, which included diabetes, hypertension, albumin, LDL cholesterol, triglycerides, serum magnesium, amiodarone use, and neoplastic disease.

### 4.3. Pathophysiological Links of Hypothyroidism in HD Patients

Multiple factors contribute to hypothyroidism in HD, including inflammation, malnutrition, anemia, dialysis type and duration, heparin, phosphate binders (notably sevelamer), roxadustat, and vitamin D deficiency [21,27,46,47]. Inflammatory syndrome is frequently observed in HD patients and the specific biomarkers evaluated across various studies confirm the pro-inflammatory status characteristic of this population group [48,49,50]. The inflammatory syndrome, assessed by CRP, was subclinical present across the entire patient cohort. However, patients with hypothyroidism did not exhibit significantly higher CRP levels, and inclusion of CRP in the multivariate logistic regression did not support its role as a predictor of hypothyroidism. This finding remained valid for both autoimmune thyroiditis and subclinical hypothyroidism.

Protein malnutrition has a substantial impact on the development of hypothyroidism by reducing hormone synthesis, binding proteins and enzymes involved in hormone metabolism. In our patients, malnutrition was assessed via serum albumin levels. Hypothyroid patients exhibited significantly lower serum albumin compared to euthyroid individuals. Moreover, hypoalbuminemia emerged as a strong predictor of hypothyroidism in the logistic regression model.

Anemia contributes to hypothyroidism through multiple mechanisms: decreased oxygen delivery to the thyroid, leading to reduced hormone synthesis; exacerbation of malnutrition through appetite loss; decreased treatment adherence affecting quality of life; and use of medications that may induce hypothyroidism. In thyroid insufficiency, anemia can result from several factors: impaired hemoglobin synthesis, iron deficiency from reduced intestinal absorption or menorrhagia, folate deficiency from decreased absorption, and vitamin B12–related megaloblastic anemia caused by autoimmune mechanisms, especially in Hashimoto’s thyroiditis. In our cohort, mean hemoglobin levels were within therapeutic targets recommended by current guidelines and no significant differences were observed between hypothyroid and non-hypothyroid patients, regardless of the underlying etiology.

Patients on HD exhibit a distinct lipid profile, characterized by elevated triglycerides and LDL cholesterol, along with reduced HDL cholesterol, a pattern that promotes atherogenesis. The relationship between dyslipidemia and hypothyroidism may be bidirectional. On one hand, thyroid hormone deficiency alters lipid metabolism by increasing the synthesis of atherogenic lipids, including ApoB-containing lipoproteins, through inhibition of HMG-CoA reductase, increased intestinal cholesterol absorption and reduced clearance, such as impaired reverse cholesterol transport. Hypothyroidism has also been associated with increased ectopic fat deposition in the liver, a strong predictor of future dyslipidemia, hyperglycemia, metabolic dysfunction-associated fatty liver disease (MAFLD) and elevated cardiometabolic risk. On the other hand, dyslipidemia and obesity may contribute to the development of hypothyroidism [51]. Consistent with data from the literature, hypothyroid patients in our cohort exhibited significantly higher triglyceride and LDL cholesterol levels compared to euthyroid patients, with no differences observed in HDL cholesterol. This lipid profile persisted in cases of subclinical hypothyroidism but was not observed in patients with autoimmune thyroiditis or in those with hypothyroidism due to amiodarone usage.

### 4.4. The Clinical Impact of Hypothyroidism in HD Patients

The major impact of hypothyroidism in HD patients concerns cardiovascular pathology. The consequences of thyroid hormone deficiency on the cardiovascular system include exacerbation of endothelial dysfunction, promotion of atherogenesis, increased incidence of adverse cardiovascular events, systolic and diastolic cardiac dysfunction and worsening of pre-existing heart failure [16,44,52,53].

Studies investigating the impact of hypothyroidism on vascular calcifications in HD patients have demonstrated increased Agatston scores and coronary artery calcium (CAC) volumes in these individuals [53]. A prospective study of 438 HD patients with low T3 syndrome confirmed the cardiovascular consequences of this condition, correlating with pre-existing cardiovascular impairment and elevated proBNP levels. However, the study did not demonstrate an independent predictive value of low T_3_ for the occurrence of major adverse cardiovascular events (MACE) [32].

In our study, compared with euthyroid patients, those with hypothyroidism exhibited higher rates of persistent atrial fibrillation and greater need for interventional cardiology, despite similar incidence of CAD, acute heart failure, hypertension, and stroke. These differences were absent in autoimmune thyroiditis but persisted in subclinical hypothyroidism. No differences were observed regarding cardiovascular mortality in hypothyroid patients.

An increased erythropoietin (EPO) requirement has been reported in the literature as a potential consequence of hypothyroidism [54]. In our cohort, erythropoietin (EPO) requirements were similar between groups. All-cause mortality did not differ significantly; however, since neoplastic disease was more frequent in hypothyroid patients, cancer-related deaths were significantly higher in linear regression analyses, although this association was not confirmed in the multivariate regression model.

The benefits of timely diagnosis and treatment of hypothyroidism in patients undergoing HD primarily target cardiovascular risk. Vascular fragility predisposes these patients to frequent coronary and cerebrovascular events, while the coexistence of hypothyroidism-related dyslipidemia, endothelial dysfunction, increased arrhythmia prevalence and a high incidence of heart failure further amplifies their vulnerability [55].

The strengths of our study include confirmation of literature data regarding the prevalence of hypothyroidism in HD patients, particularly among females and its strong association with hypoalbuminemia and amiodarone use. Our findings also demonstrate the negative cardiac impact of hypothyroidism, reflected by an increased need for interventional cardiology. The observed association with neoplastic disease warrants further investigation in larger, dedicated studies. Consequently, we consider it useful to integrate annual routine screening for hypothyroidism in HD patients, particularly those with protein malnutrition, severe dyslipidemia, or undergoing amiodarone therapy. A multidisciplinary management approach may offer a comprehensive strategy to mitigate the risks associated with this condition. Moreover, the results of our study, by delineating the specific profile of HD patients with hypothyroidism—predominantly female, overweight, with low albumin, elevated cholesterol and triglycerides, and a higher incidence of cardiac arrhythmias—may serve as a starting point for developing a set of recommendations in diagnostic and treatment guidelines for hypothyroidism tailored to this patient population, recommendations that are not currently included in existing clinical practice guidelines.

This study has several limitations. Its retrospective design, based on pre-existing medical records, precludes establishing causal relationships. A fundamental limitation of the study was the retrospective inclusion of hypothyroid patients receiving thyroxine supplementation, which altered biochemical and anthropometric parameters as well as potential cardiovascular outcomes, potentially reducing the sensitivity of hypothyroidism predictors in this cohort. The data we obtained regarding hormone replacement therapy in our patients were insufficient to perform a meaningful analysis based on this parameter. Being a single-center study, the findings may not be generalizable to other HD populations and selection bias cannot be entirely excluded. Restricting inclusion to patients with a dialysis vintage of over 12 months may introduce selection and survivor biases, potentially underestimating the prevalence of hypothyroidism and related risk factors. Additionally, the sample size may limit the statistical power to detect less common predictors and residual confounding—such as lifestyle factors, residual kidney function, and dialysis prescription—cannot be ruled out. Nonetheless, the comprehensive evaluation of clinical, laboratory, and dialysis-related parameters enhances the robustness of our findings, while acknowledging the inherent limitations of a retrospective study design.

## 5. Conclusions

Hypothyroidism is a common condition among patients undergoing HD. Its occurrence has been associated with HD duration, female sex, hypoalbuminemia, overweight, dyslipidemia, amiodarone administration, and possibly with reduced HD efficiency. Evaluating the impact of consumptive diseases, predominantly malignancies, on the development of this condition represents a potential research hypothesis. Considering its influence on cardiovascular pathology—through the exacerbation of endothelial dysfunction, myocardial alterations, and its association with arrhythmias and heart failure—an individualized approach to this condition in HD patients is warranted within the context of diagnostic and therapeutic guidelines for hypothyroidism in general.

## Figures and Tables

**Table 1:** Clinical and laboratory characteristics of our patients.

Parameter	Entire Cohort 282 M ± SD/%	Hypothyroidism 66 (23.4%) M ± SD/%	Euthyroidism 216 (76.55%) M ± SD/%	*p*
Age	64.273 ± 13.823	63.712 ± 14.103	64.444 ± 14.13	0.707
HD vintage	6.727 ± 5.748	6.985 ± 6.487	6.648 ± 5.717	0.678
Sex F	131 (46.45%)	42 (63.63%)	89 (41.2%)	<0.001
AVF	196 (69.5%)	42 (63.63%)	154 (71.29%)	0.237
BMI (kg/m^2^)	26.292 ± 6.171	27.856 ± 6.216	25.759 ± 6.080	0.017
CGN	100 (35.46%)	21 (301.81%)	79 (36.57%)	0.450
VN	62 (21.98%)	15 (22.72%)	47 (21.76%)	0.896
DN	51 (18.08%)	11 (16.66%)	40 (18.51%)	0.794
PKD	21 (7.45%)	7 (10.66%)	14 (6.48%)	0.273
PTx	39 (13.83%)	12 (18.18%)	27 (12.5%)	0.242
TSH (μUI/mL)	5.257 ± 13.819	10.267 ± 21.244	2.065 ± 1.209	<0.001
fT_4_ (ng/dL)	0.982 ± 0.349	0.965 ± 0.267	1.009 ± 0.453	0.284
fT_3_ (pg/mL)	2.030 ± 0.713	1.877 ± 0.604	2.289 ± 0.814	0.0064
Hb (g/dL)	10.605 ± 1.451	10.439 ± 1.311	10.656 ± 1.490	0.228
Albumin (g/dL)	3.681 ± 0.496	3.534 ± 0.547	3.725 ± 0.471	0.006
hs-CRP mg/dL	29.041 ± 43.138	30.111 ± 38.465	28.688 ± 44.657	0.817
T-Cholesterol (mg/dL)	158.996 ± 47.265	167.5 ± 55.291	151.0 ± 43.848	0.042
LDL-cholesterol (mg/dL)	102.341 ± 39.561	114.375 ± 42.096	95.834 ± 36.697	0.002
HDL-cholesterol (mg/dL)	38.244 ± 16.033	36.402 ± 10.074	39.593 ± 19.214	0.231
Triglycerides (mg/dL)	143.007 ± 61.053	175.258 ± 53.984	133.061 ± 59.761	<0.001
iPTH (pg/mL)	407.146 ± 435.548	412.07 ± 436.054	405.425 ± 436.702	0.921
Calcium (mg/dL)	8.836 ± 0.832	8.715 ± 0.871	8.873 ± 0.819	0.182
Phosphate (mg/dL)	4.849 ± 1.728	4.819 ± 1.522	4.795 ± 1.794	0.873
Kt/V	1.575 ± 0.308	1.512 ± 0.289	1.596 ± 0.312	0.053
Epo dose	88.005 ± 210.914	78.772 ± 115.44	89.667 ± 224.2	0.841
Amiodarone use	20	13 (19.69%)	7 (3.24%)	<0.001
Death	160	38 (57.57%)	122 (56.48%)	0.875

Abbreviations: M—mean; SD—standard deviation; HD—hemodialysis; AVF—arteriovenous fistula; BMI—body mass index; CGN—chronic glomerulonephritis; VN—vascular nephropathy; DN—diabetic nephropathy; PKD—polycystic kidney disease; PTx—parathyroidectomy; TSH—thyroid-stimulating hormone; fT_3_—free T_3_; fT_4_—free T_4_; Hb—hemoglobin; hs-CRP—high sensitive C reactive protein; iPTH—intact parathormone; Kt/V—index of dialysis adequacy.

**Table 3:** Predictors for hypothyroidism in HD in multivariable logistic regression.

Predictors for Hypothyroidism	z	*p*	Odds Ratio	95% Confidence Interval
gender	3.242	0.001	3.848	1.704–8.693
BMI (kg/m^2^)	2.162	0.031	1.072	1.007–1.146
albumin	−2.051	0.040	0.412	0.177–0.962
LDL-cholesterol	2.675	0.007	1.014	1.004–1.024
triglycerides	2.445	0.014	1.009	1.002–1.016
amiodarone usage	2.770	0.006	6.698	1.744–25.722
Neoplastic-related death	1.880	0.060	5.887	0.927–37.381
Atrial fibrillation	0.295	0.768	1.143	0.471–2.776

Legend: HD—hemodialysis; BMI—body mass index.

**Table 4:** Predictors for hypothyroidism in HD patients. Model Fit Measures. Predictive Measures.

**Model**	**Deviance**	**AIC**	**R^2^_McF_**	**Specificity**	**Sensitivity**	**AUC**
	154.800	172.800	0.271	0.857	0.603	0.829

Abbreviations: AIC—Akaike Information Criterion; R^2^_McF_—McFadden’s R^2^; AUC—Area Under the Curve.

**Table 5:** Autoimmune thyroiditis characteristics in HD patients.

Parameter	Hypothyroidism 66 M ± SD/%	Autoimmune Thyroiditis 15 (22.72%) M ± SD/%	Non-Autoimmune Hypothyroidism 51 (77.27%) M ± SD/%	*p*
Age	63.712 ± 14.103	64 ± 13.836	63.627 ± 12.826	0.923
HD vintage	6.985 ± 6.487	4.533 ± 2.949	7.706 ± 7.066	0.096
Sex F	42 (63.63%)	9 (60%)	33 (64.7%)	0.739
AVF	42 (63.63%)	10 (66.66%)	32 (62.74%)	0.781
BMI (kg/m^2^)	27.856 ± 6.216	31.067 ± 5.951	26.912 ± 6.026	0.022
CGN	21 (31.81%)	7 (46.66%)	14 (27.45%)	1
VN	15 (22.72%)	3 (20%)	12 (23.52%)	0.774
DN	11 (16.16%)	3 (20%)	8 (15.68%)	0.694
PKD	7 (10.6%)	0	7 (13.72%)	0.129
PTx	12 (18.18%)	1 (6.66%)	11 (21.56%)	0.188
TSH (μUI/mL)	10.267 ± 21.244	5.503 ± 4.472	11.696 ± 23.973	0.326
Hb (g/dL)	10.439 ± 1.311	10.207 ± 1.453	10.507 ± 1.274	0.439
Albumin (g/dL)	3.534 ± 0.547	3.433 ± 0.482	3.564 ± 0.566	0.417
hs-CRP (mg/dL)	30.111 ± 38.465	28.067 ± 39.834	30.712 ± 38.438	0.817
T-Chol (mg/dL)	167.5 ± 55.291	164.933 ± 43.051	170.735 ± 58.855	0.725
LDL-chol (mg/dL)	114.375 ± 42.096	121.133 ± 38.469	112.320 ± 43.283	0.481
HDL-chol (mg/dL)	36.402 ± 10.074	39.267 ± 10.292	35.506 ± 9.944	0.210
Triglycerides (mg/dL)	175.258 ± 53.984	163.53 ± 48.494	178.70 ± 55.469	0.343
iPTH (pg/mL)	412.07 ± 436.054	402.214 ± 207.58	415.279 ± 490.044	0.923
Calcium (mg/dL)	8.715 ± 0.871	8.939 ± 0.764	8.654 ± 0.895	0.281
P (mg/dL)	4.819 ± 1.522	4.566 ± 1.210	4.892 ± 1.603	0.484
Kt/V	1.512 ± 0.289	1.459 ± 0.253	1.527 ± 0.299	0.424
Hypertension	60 (90.90%)	14 (93.33%)	46 (90.19%)	0.710
Diabetes	17 (25.75%)	5 (33.33%)	12 (23.52%)	0.445
CAD	21 (31.81)	4 (26.66%)	17 (33.33%)	1
Malignancies	17 (25.75%)	4 (26.66%)	13 (25.49%)	0.927
Amiodarone use	13 (19.69%)	2 (13.33%)	11 (21.56%)	0.465
CADR	12 (18.18%)	3 (20%)	9 (17.64%)	0.823
Paroxysmal AF	17 (25.75%)	5 (33.33%)	12 (23.52%)	0.497
Permanent AF	22	4 (26.66%)	18 (35.29%)	0.473
Death	38 (57.57%)			
CV death	13 (19.69%)	1 (6.66%)	12 (23.52%)	0.149
Neoplastic death	7 (10.6%)	0	7 (13.72%)	0.129

Legend: HD—hemodialysis; F—female; AVF—arteriovenous fistula; BMI—body mass index; CGN—chronic glomerulonephritis; VN—vascular nephropathy; DN—diabetic nephropathy; PKD—polycystic kidney disease; PTx—patathyroidectomy; TSH—thyroid stimulating hormone; Hb—hemoglobin; hs-CRP—high sensitive C reactive protein; T-chol—total cholesterol; LDL-Chol—LDL-cholesterol; HDL-Chol—HDL-cholesterol; iPTH—intact parathormone; P—phosphate; Kt/V—index of dialysis adequacy; CAD—coronary artery disease; CADR—coronary artery disease revascularization; AF—atrial fibrillation; CV—cardiovascular.

**Table 6:** Main characteristics in HD subclinical hypothyroidism patients.

Parameter	Subclinical Hypothyroidism 21 (31.81%) M ± SD/%	Euthyroidism 216 (76.55%) M ± SD/%	*p*
Age	63.286 ± 14.364	64.444 ± 14.13	0.360
HD vintage (years)	10.476 ± 7.910	6.648 ± 5.717	<0.001
Sex (F)	13 (61.9%)	89 (41.2%)	0.109
AVF	15 (71.42%)	154 (71.29%)	0.810
BMI (kg/m^2^)	25.643 ± 5.325	25.759 ± 6.080	0.466
CGN	7 (33.33%)	79(36.57%)	0.954
VN	4 (19.04%)	47 (21.75%)	0.991
DN	2 (9.52%)	40 (18.51%)	0.464
PKD	3 (14.28%)	14 (6.48%)	0.378
PTx	5 (23.8%)	27 (12.5%)	0.265
TSH (μUI/mL)	4.647 ± 1.785	2.065 ± 1.209	<0.00001
Hb (g/dL)	10.519 ± 1.086	10.656 ± 1.490	0.341
Albumin (g/dL)	3.51 ± 0.618	3.725 ± 0.471	0.026
hs-CRP (mg/dL)	28.076 ± 29.083	28.688 ± 44.657	0.475
T-Chol (mg/dL)	176.950 ± 67.966	151.0 ± 43.848	0.025
LDL-chol (mg/dL)	117.905 ± 51.103	95.834 ± 36.697	0.0091
HDL-chol (mg/dL)	35.465 ± 2.330	39.593 ± 19.214	0.177
Triglycerides (mg/dL)	178.048 ± 11.436	133.061 ± 59.761	0.0005
iPTH (pg/mL)	451.263 ± 116.71	405.425 ± 436.702	0.335
Calcium (mg/dL)	8.797 ± 0.179	8.873 ± 0.819	0.343
P (mg/dL)	5.143 ± 0.44	4.795 ± 1.794	0.248
Kt/V	1.61 ± 0.068	1.596 ± 0.312	0.424
Mg (mg/dL)	1.952 ± 0.745	2.133 ± 0.053	0.299
Epo dose	139.883 ± 74	89.667 ± 224.2	0.296
Hypertension	18 (85.71%)	205 (94.9%)	0.222
Diabetes	3 (14.28%)	72 (33.33%)	0.122
CAD	9 (42.85%)	70 (32.4%)	0.467
Neoplastic diseases	3 (14.28%)	26 (12.03%)	0.961
Amiodarone use	5 (23.8%)	7 (3.24%)	0.0003
CADR	3 (14.28%)	21 (9.72%)	0.777
Paroxysmal AF	5 (23.8%)	80 (37.03%)	0.332
Permanent AF	11 (52.38%)	43 (19.90%)	0.0018
CV death	8 (38.09%)	65 (30.09%)	0.609
Neoplastic death	3 (14.28%)	4 (1.85%)	0.011

Legend: HD—hemodialysis; F—female; AVF—arteriovenous fistula; BMI—body mass index; CGN—chronic glomerulonephritis; VN—vascular nephropathy; DN—diabetic nephropathy; PTx—parathyroidectomy; PKD—polcystic; TSH—thyroid stimulating hormone; Hb—hemoglobin; hs-CRP—high sensitive C reactive protein; T-chol—total cholesterol; LDL-Chol—LDL-cholesterol; HDL-Chol—HDL-cholesterol; iPTH—intact parathormone; P—phosphate; Kt/V—index of dialysis adequacy; Mg—magnesium; CAD—coronary artery disease; CADR—coronary artery disease revascularization; AF—atrial fibrillation; CV—cardiovascular.

**Table 7:** Predictors for subclinical hypothyroidism in multivariable regression model.

Predictor	*p*	Odds Ratio	95% Confidence Interval
HD vintage	0.017	1.093	1.016–1.175
albumin	0.06	0.380	0.139–1.041
Amiodarone usage	<0.001	13.649	3.536–52.691

Legend: HD = hemodialysis.

**Table 8:** Predictors for subclinical hypothyroidism. Model Fit Measures. Predictive Measures.

**Model**	**AIC**	**R^2^_McF_**	**Accuracy**	**Specificity**	**AUC**
	108.768	0.172	0.936	0.995	0.748

Legend: AIC = Akaike Information Criterion; R^2^_McF_ = McFadden’s R^2^; AUC = Area Under the Curve.

**Table 9:** Main characteristics of the patients using amiodarone.

Parameter	Amiodarone Usage 20	Non-Amiodarone Usage 257	*p*-Value
Age	68.200 ± 11.143	64.031 ± 13.875	0.191
HD vintage	7.2 ± 5.899	6.599 ± 5.598	0.645
gender F	9 (45%)	120	0.884
AVF	16 (80%)	176	0.282
BMI (kg/m^2^)	28.5 ± 5.066	26.056 ± 6.240	0.025
Hypertension	19 (95%)	242 (94.16%)	0.955
Diabetes	6 (30%)	81 (31.51%)	0.861
CAD	7 (35%)	82 (31.0%)	0.803
Malignancies	2 (10%)	41 (15.95%)	0.463
CGN	4 (20%)	93 (36.18%)	0.138
VN	8 (40%)	54 (21.01%)	0.052
DN	2 (10%)	47 (18.28%)	0.608
PKD	2 (10%)	19 (7.39%)	0.679
PTx	3 (15%)	35 (13.61%)	0.863
TSH (μUI/mL)	6.139 ± 4.919	5.129 ± 14.883	0.769
Hb (g/dL)	10.319 ± 1.031	10.642 ± 1.476	0.337
Albumin (g/dL)	3.526 ± 0.501	3.694 ± 0.498	0.147
hs-CRP (mg/dL)	24.725 ± 37.855	29.097 ± 43.293	0.662
T-Chol (mg/dL)	147.5 ± 40.433	152.0 ± 47.372	0.334
LDL-chol (mg/dL)	96.5 ± 41.458	102.6 ± 42.068	0.608
HDL-chol (mg/dL)	37.917 ± 10.022	40.199 ± 18.288	0.672
Triglycerides (mg/dL)	148.647 ± 87.491	149.265 ± 95.597	0.980
iPTH (pg/mL)	554.6 ± 637.28	397.141 ± 420.245	0.181
Calcium (mg/dL)	8.970 ± 0.952	8.822 ± 0.830	0.448
P (mg/dL)	5.170 ± 1.902	4.827 ± 1.726	0.398
Kt/V	1.516 ± 0.289	1.580 ± 0.312	0.398
Hypothyroidism	13 (65%)	7 (2.72%)	<0.001
Paroxysmal AF	12 (60%)	85 (33.07%)	0.020
Permanent AF	8 (40%)	54 (21.01%)	0.061
CV death	7 (35%)	69 (26.84%)	0.431
Neoplastic death	0	11 (4.28%)	0.345

Legend: HD—hemodialysis; F—female; AVF—arteriovenous fistula; BMI—body mass index; CGN—chronic glomerulonephritis; VN—vascular nephropathy; DN—diabetic nephropathy; PKD—polycystic kidney disease; PTx—patathyroidectomy; TSH—thyroid stimulating hormone; Hb—hemoglobin; hs-CRP—high sensitive C reactive protein; T-chol—total cholesterol; LDL-Chol—LDL-cholesterol; HDL-Chol—HDL-cholesterol; iPTH—intact parathormone; P—phosphate; Kt/V—index of dialysis adequacy; CAD—coronary arthery disease; AF—atrial fibrillation; CV—cardiovascular.

## Data Availability

The original contributions presented in this study are included in the article. Further inquiries can be directed to the corresponding authors.
